# Advancing the Physicochemical Properties and Therapeutic Potential of Plant Extracts Through Amorphous Solid Dispersion Systems

**DOI:** 10.3390/polym16243489

**Published:** 2024-12-14

**Authors:** Arif Budiman, Nur Parida Mahdhani Hafidz, Raden Siti Salma Azzahra, Salma Amaliah, Feggy Yustika Sitinjak, Agus Rusdin, Laila Subra, Diah Lia Aulifa

**Affiliations:** 1 Department of Pharmaceutics and Pharmaceutical Technology, Faculty of Pharmacy, Universitas Padjadjaran, Jl. Raya Bandung-Sumedang Km. 21, Bandung 45363, Indonesia; salma19008@mail.unpad.ac.id (S.A.); feggy24001@mail.unpad.ac.id (F.Y.S.); agusrusdin@gmail.com (A.R.); 2 Department of Pharmaceutical Analysis and Medicinal Chemistry, Faculty of Pharmacy, Universitas Padjadjaran, Jl. Raya Bandung-Sumedang Km. 21, Bandung 45363, Indonesia; nur21024@mail.unpad.ac.id (N.P.M.H.); rsiti21001@mail.unpad.ac.id (R.S.S.A.); diah.lia@unpad.ac.id (D.L.A.); 3 Department of Pharmacy, Faculty of Bioeconomic, Food and Health Sciences, Universiti Geomatika Malaysia, Kuala Lumpur 54200, Malaysia; laila@geomatika.edu.my

**Keywords:** medicinal plant extracts, amorphous solid dispersion, polymer, dissolution, oral bioavailability

## Abstract

Plant extracts demonstrate significant potential as a rich source of active pharmaceutical ingredients, exhibiting diverse biological activities and minimal toxicity. However, the low aqueous solubility of extracts and their gastrointestinal permeability, as well as their poor oral bioavailability, limit clinical advancements due to drug delivery problems. An amorphous solid dispersion (ASD) delivers drugs by changing an active pharmaceutical ingredient (API) into an amorphous state to increase the solubility and availability of the API to the body. This research aimed to analyze and summarize the successful advancements of ASD systems derived from plant extracts, emphasizing characterization and the effects on dissolution and pharmacological activity. The results show that ASD systems improve phytoconstituent dissolution, bioavailability, and stability, in addition to reducing dose and toxicity. This research demonstrates the significance of ASD in therapeutic formulations to augment the pharmacological activities and efficacy of medicinal plant extracts. The prospects indicate promising potential for therapeutic applications utilizing ASD systems, alongside medicinal plant extracts for clinical therapy.

## 1. Introduction

Plant extracts demonstrate significance as a rich source of active pharmaceutical ingredients, accounting for 40% of all approved therapeutic drugs [1,2]. Plants are anticipated to contribute substantially to the discovery of novel drug candidates among all natural resources. About 25% of all products sanctioned by the FDA are derived from botanical sources [3,4]. Despite the absence of a definitive pharmacological mechanism and meticulously conducted clinical trials, plant-derived natural products remain a principal source of pharmaceuticals for the prevention and treatment of pathological disorders for approximately 80% of the global population.

The ability of plants to provide various pharmacological compounds is primarily due to the structural diversity of secondary metabolites, which are largely produced for defense against herbivorous animals. Drug compounds derived from natural resources show excellent characteristics of binding to numerous targets in comparison with complicated synthesized molecules. However, the properties of being sticky, adhesive, and hygroscopic make them harder to process and turn into profitable oral formulations such as capsules and tablets. The challenges in isolating and characterizing a specific compound from an extract library, the absence of well-defined biological mechanisms, and inadequate solubility resulting in minimal bioavailability following oral administration are significant obstacles affecting the successful formulation of various phytoconstituents for oral use [5].

A natural product’s oral bioavailability is very important for its success in the market because it is used to treat diseases such as cancer, neurodegeneration, infections, and metabolic disorders, which need long treatment periods. There are several formulation methods for insoluble drugs, since herbal extracts have low water solubility, leading to limited oral bioavailability [6]. Common methods encompass modifications of crystals, amorphous transformation, self-emulsification, decreases in particle size, and alterations in pH [7,8]. Recent years have seen the documentation of various methods of improving the dissolution rate and bioavailability of extracts, including self-emulsifications, solid dispersions, and phospholipid complexations [9,10,11,12].

Amorphous solid dispersions (ASDs) have demonstrated their effectiveness as a straightforward, industry-compatible, and robust technique. In an ASD, a drug is encapsulated or molecularly dispersed within a polymeric matrix in an amorphous form [13]. The amorphous state of a material exhibits significantly higher internal free energy than the crystalline state [14,15]. Upon contact with dissolving fluids, the polymer matrix dissolves, resulting in the swift release of drugs. Compared with the crystalline counterparts, this resulted in a substantial improvement in drug dissolution [16].

Amorphous materials, due to their significant instability, typically revert to a crystalline state, which is more stable and less soluble in water. Consequently, the amorphous state must be stabilized by limiting molecular mobility within the polymer matrix. The drug is stabilized by weak attractive forces or encapsulated within the polymer matrix. The formation of an ASD improves the solubility and essential physicochemical properties of an extract, such as its flowability and compressibility [17].

The research interest in ASDs of plant extracts originated in the protection of bioactive substances derived from plant extracts while improving their dissolution, bioavailability, and pharmacological activity. Although several studies have been published on the utilization of a polymer matrix for plant extracts, there is a lack of articles on the explanation and deep analysis of plant extracts in terms of their pharmacological properties. Therefore, this research aimed to present knowledge on the utilization of ASD systems to improve the efficacy of plant extracts. Insights are provided into the elucidation of ASD formulations made from plant extracts and their effects on dissolution and pharmacological activity. Journal articles on ASD systems were obtained from Google Scholar, Scopus, and Pubmed, and all of them were published within the last 10 years. This provides a foundation for directing future research on the effective application of ASDs with plant extracts, leading to novel therapeutic methods for addressing various disorders.

## 2. Materials and Methods

This study was based on the literature sourced from Google Scholar, Scopus, and PubMed databases using specific keywords: “amorphous solid dispersion” and “medicinal plant extract”. Meanwhile, opinions, studies published in languages other than English, and studies addressing unrelated subjects were excluded from consideration.

## 3. Medicinal Plant Extracts

Medicinal plant extracts produce chemical compounds with pharmacological or biological properties. Herbal medicines are natural resources that can prevent and treat diseases. These plants play a crucial and irreplaceable role in drug discovery and development [18]. Herbal medicines are commonly formulated for oral administration, such as through droplet pills, capsules, and tablets, as well as for parenteral administration, such as in injectable formulations [19].

The advancement of modern pharmacology and healthcare procedures has been influenced by medicinal plants.Throughout history, several cultures have recognized and used the therapeutic properties of plants. Herbal therapies have been adopted since antiquity, as evidenced by archeological results dating back to the Paleolithic epoch of 60,000 years ago. The Sumerians, who collected plant inventories, left written records of herbal therapies dating back over 5000 years. Ancient societies such as the Egyptians, Greeks, and Romans relied largely on medicinal plant extracts for medicinal purposes [20]. Recently, patented medicinal plant extracts have shown therapeutic advantages comparable with those of modern medications in clinical research [19].

Medicinal plant extracts have contributed to drug discovery for cancer, infectious diseases, cardiovascular disorders, multiple sclerosis, and minor illnesses such as headaches, stomach aches, fractures, and sprains [20,21]. Herbal medicines have unique properties that provide advantages for the drug discovery process when compared with traditional synthetic compounds. The extracts are distinguished by their vast scaffold variety and structural complexity, serving as a key source of oral medications beyond Lipinski’s rule of five. Herbal medications are evolutionarily refined to execute particular biological functions, such as regulating endogenous defense mechanisms and interacting with other species, underscoring their importance concerning infectious diseases and cancer (Table 1). The application of conventional medicine provides insights into its efficacy and safety. The pool of herbal medicines contains more bioactive molecules covering a larger chemical region than standard synthetic small-molecule libraries [21]. According to Chaachouay and Zidane (2024), natural compounds derived from plants can be positioned to maintain the importance of viable sources for developing innovative treatments and methods [22].

Numerous herbal medications and extracts exhibit diminished or negligible in vivo efficacy due to inadequate lipid solubility or inappropriate molecular size, leading to poor absorption and bioavailability. Diverse constituents of an extract enhance the synergistic efficacy of the extract and the treatment. In this context, purification and separation may result in a partial reduction in specific activity due to the elimination of chemically related substances that contribute to the primary components. The chemical complexity of an extract is crucial for the bioavailability of the active constituents. The majority of plant constituents are soluble in water. The primary issue regarding reduced bioavailability is the inability to traverse the lipid membranes of the intestines. Bioavailability can be enhanced through various innovative delivery systems, including liposomes, marinosomes, niosomes, and lipid-based systems, to improve the release rate and the ability to traverse biomembranes. Numerous medicinal plant extracts, which include phytoconstituents such as flavonoids, tannins, terpenoids, and polyphenolics, exhibit high solubility in water, yet demonstrate poor absorption due to their large, multi-ring molecular structures (500–4000 Da) [42].

The activities of medicinal plant extracts encounter several issues, including physicochemical factors resulting in inadequate and inconsistent solubility, chemical instability in acidic pH, metabolic degradation, and insufficient permeation across the intestinal barrier [43].

1 Solubility

Active compounds in herbs often have poor solubility in water or organic solvents, which hinders the ability to dissolve and be absorbed effectively by the body. Lipophilic compounds are not soluble in water and may require special solvents or formulations to improve solubility [44].

2 Chemical Stability

Many herbal compounds have low chemical stability, with the ability to degrade or lose biological activity when exposed to extreme pH or oxidation. In the digestive system, the acidic pH of the stomach breaks down or alters active compounds before absorption into the bloodstream [45].

3 Metabolism

Active herbal compounds are absorbed into the body and metabolized by enzymes in the liver and digestive system. This process can convert the compounds into less active forms, reducing the therapeutic efficacy [46].

4 Intestinal Permeability

Active herbal compounds must pass through the intestinal wall to enter the bloodstream and exert their effects throughout the body. However, some compounds may have difficulty crossing the intestinal cell membranes, limiting the level of absorption by the body [47].

## 4. Amorphous Solid Dispersions (ASDs)

An ASD is an effective approach that is frequently employed to enhance the oral bioavailability of limited water-soluble pharmaceuticals. Such a system comprises an amorphous active pharmaceutical ingredient stabilized by a polymeric matrix for enhanced stability. The choice of a suitable polymer carrier enhances the dissolution rate, solubility, and solid-state physical stability. A polymer carrier facilitates the transformation of crystalline drugs into amorphous forms and concurrently stabilizes amorphous solid dispersions (ASDs) by restricting molecular mobility and elevating the glass transition temperature (Tg) [48]. The application of ASDs for oral drug delivery has demonstrated enhanced in vitro performance and in vivo bioavailability in animal studies. Nevertheless, applications in drug development are infrequent due to the insufficient comprehension of the mechanisms governing drug release and absorption in vivo [49]. 

### 4.1. Factors Affecting Dissolution

Factors affecting ASD dissolution are deduced from mechanical concerns. Aside from knowing the disintegration of an ASD, various factors influence the transition from solid to dissolved form.

1 Effect of the Drug Load

Higher drug doses are preferable in formulations designed for low pill loads. However, increased drug loading has a direct impact on dissolution and the characteristics of ASDs [49]. At lower drug loading levels, drug and polymer release were consistent, with no evidence of crystallization. At higher drug loading levels, drug crystallization occurred after the polymer was released from the ASD, causing delayed dissolution [50,51,52]. Higher drug loading may affect controlled (congruent) dissolution, leading to drug release and failure to achieve the desired supersaturation effect.

2 Effect of the Homogeneity of the Molecular Distribution in an ASD

The molecular arrangement within an ASD is crucial, necessitating a wholly amorphous state for optimal dissolution. The existence of crystalline domains alters particle behavior. Amorphous ASDs generate colloids devoid of a crystalline phase. Concurrently, colloids exhibiting residual crystallinity or amorphous drug domains in the solid state demonstrate phase separation within the particles, leading to significant crystal growth. The drug and polymer are released simultaneously when uniformly distributed within the polymer matrix [53,54]. An inhomogeneous distribution results in the polymer being released more rapidly than the drug. Substantial quantities of drug–polymer mixtures enhance the apparent solubility of amorphous solid dispersions during dissolution.

3 Effects of Wetting Characteristics

Pre-wetting ASD particles prior to dissolution is essential, as this significantly influences the dissolution kinetics. Accelerated wetting kinetics are correlated with dissolution rates [55]. Nonetheless, ASDs did not exhibit the anticipated behavior regarding the components. Unlike physical mixtures of active pharmaceutical ingredients (APIs) and polymers, the wetting behavior cannot be elucidated using the individual components [56].

4 Effects of Drug–Polymer Interactions

The impact of drug–polymer interactions on dissolution performance was analyzed. The release of drugs and polymers was consistent in systems exhibiting substantial interactions and uniform amorphous solid dispersion mixing, which was characterized by dissolution-controlled release. Moreover, diminished drug release rates were observed in systems exhibiting restricted polymer–drug interactions [57]. Intense interactions between the drug and carrier impede the release rate. From a conceptual standpoint, this interaction is anticipated for carrier-mediated release, wherein robust contacts may diminish effective diffusion [58]. A comprehensive investigation of the relationship between drug–polymer interactions and dissolution properties is necessary to elucidate the molecular mechanism of medications using ASDs [49]. This is significant in formulation development, as the interactions may be anticipated or quantified, potentially facilitating early predictions.

5 Effects of Surfactants

Surfactants have been used to improve dissolution rates when embedded in ASDs with deleterious effects [59]. Meng et al. (2019) found that the use thereof increased the dissolution and supersaturation effects. Although surfactants enhanced the dissolution properties of ASDs, additional investigation of the influence of colloidal formation on drug absorption is required [60].

6 Extrinsic Factors

Luo et al. (2019) indicate that various dissolution mechanisms may be pertinent based on the pH of the medium [61]. Polymer degradation seems pertinent at low pH, whereas swelling has been observed at elevated pH levels. The presence of surfactants directly influences the dissolution performance through ionic strength [62]. Fotaki et al. (2014) assert that examining the dissolution behavior in biorelevant media is essential for forecasting in vivo outcomes. Although further investigation of the impact of dissolution media and their role in ASD development is necessary, integrating experiments across various media may be the most effective approach for differentiating between ASD formulations and elucidating their behavior under physiological conditions [63].

### 4.2. Mechanism of ASDs for Increasing Bioavailability

Drug absorption mechanisms have been investigated in various studies. Solution-state spontaneous dissolution transpires when amorphous solid dispersions interact with an aqueous medium. Furthermore, APIs generate micelles, crystalline, or amorphous suspensions, and drug-enriched particles. Several references indicated that the formation of a colloidal system facilitated the intestinal absorption of dissolved active pharmaceutical ingredients (APIs) [64]. Absorption is a multi-step process comprising (i) the dissolution of an ASD, (ii) drug uptake from the dissolved API, and (iii) equilibrium in the dissolved solutions. Solution states are defined as API and API-supersaturated solutions at peak concentrations, and these are referred to as crystalline stability and amorphous solubility, respectively [65]. Crystalline solubility results from a thermodynamic equilibrium between an excess of crystalline solid and dissolved active pharmaceutical ingredients in a dissolving medium, with the structure representing the most stable polymorph. An ASD adheres to the same principle in dissolution, yet the equilibrium is metastable. This phase forms spontaneously when a supersaturated drug solution exceeds the amorphous solubility. Amorphous liquid phase separation refers to the spontaneous crystallization of drug-rich particles into a metastable state. The amorphous form of a drug possesses greater energy than its crystalline counterpart. Furthermore, amorphous materials exhibit superior apparent solubility than that of their crystalline counterparts, which is attributable to their elevated energy state and disordered structure. The amorphous state possesses elevated thermodynamic properties, including enthalpy, entropy, and Gibbs free energy. The primary factor influencing recrystallization is concurrent free energy alterations. The following equation can be utilized to determine the Gibbs free energy disparity between the amorphous and crystalline states.

Gconf = Hconf(T) + Sconf(T)

The term “configurational” denotes the distinction between the amorphous and crystalline states, along with the parameters Hconf and Sconf, which are ascertained through their correlation with heat capacity. Graeser et al. (2010) assert that an increased configuration value correlates with a more pronounced disparity between crystalline and amorphous states [66].

The formation of dissolved ASDs is essential for enhancing the solubility profile of pharmaceuticals and is directly linked to bioavailability. Craig and Simonelli innovated the carrier-based and regulated drug release of active pharmaceutical ingredients from an amorphous solid dispersion polymer combination. A viscous layer forms when the polymer remains undissolved in the solvent, thereby restricting drug release from the carrier. Likewise, when the polymer is solubilized in the solvent, the likelihood of forming a viscous layer is minimal, and dissolution is predominantly governed by the drug. The published literature provides evidence that three mechanisms are involved in the disintegration of ASDs [67,68].

ASD-based delivery systems can serve as drug carriers to address the limitations of herbal medicines, enhancing the bioavailability and bioactivity of phytochemicals. This method may serve as a promising innovative technology for enhancing the efficacy of phytotherapy using medicinal plant extracts through phytochemical constituents.

ASD systems for active herbal ingredients provide numerous benefits, such as enhanced solubility, bioavailability, pharmacological efficacy, stability, and protection against chemical and physical degradation. The objective of numerous research endeavors is the creation of an efficient and safe drug delivery system. ASDs have been employed to enhance the pharmacokinetic characteristics of various pharmaceuticals. Consequently, this method is anticipated to enhance the bioavailability and bioactivity of medicinal plant extracts [49].

## 5. Preparation of ASDs

**Figure 1:**
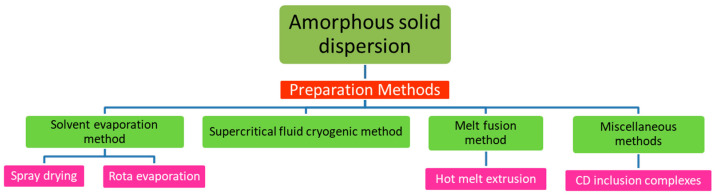
Different preparations of ASD systems.

Multiple preparation processes have been documented, as reported in Figure 1. The methods include melt fusion, such as hot melt extrusion, supercritical fluid cryogenic procedures, and solvent evaporation, namely, spray drying and rotary evaporation. The implemented methods utilize cyclodextrin-based inclusion complexes, including co-evaporation, kneading, lyophilization, microwave irradiation, electrostatic spinning, electrostatic blowing, electrospraying, and film casting. The formulations adhere to the principles of molecular solubilization mechanisms, encompassing micellar solubilization, complexation, increased porosity, or reduced particle size by utilizing various polymer-based amorphous solid dispersions [48,69].

## 6. ASD Systems for Medicinal Plant Extracts

Previous studies have reported the ASD system of medicinal plant extracts as shown in Table 2.

## 7. Characterization of ASD Systems with Medicinal Plant Extracts

### 7.1. Scanning Electron Microscopy (SEM) 

Scanning electron microscopy (SEM) is a technique that is commonly used to examine the surface and subsurface areas of ASDs. Analysis of micrographs of initial substances and extracts demonstrated pronounced morphological alterations in the powder particles post-formation. In research on an ASD with a gum resin extract, the scanning electron microscopy (SEM) images of the initial extract revealed a non-uniform shape accompanied by a rough surface texture [73]. Therefore, the ASD with the gum resin extract showed a rough surface morphology, facilitating crystalline growth during storage [73]. The results of an ASD with Polygonum Cuspidatum (PC) extract were also reported [74]. The resveratrol in the PC extract formed block crystals, while the carriers HPMCAS and P188 showed irregular and smooth morphologies, respectively. Solid dispersions lacked massive crystals, suggesting possible amorphous dispersion [74,75]. SEM images also confirmed the homogeneous dispersion of Kaempferia parviflora in polymeric carriers, and the ASD system maintained smooth and non-crystalline surfaces [75]. 

### 7.2. Differential Scanning Calorimetry (DSC)

Differential scanning calorimetry (DSC) is an extensively utilized technique for thermal analysis because the concept offers comprehensive insights into physical properties [84]. The diffractogram obtained from the pure extract showed multiple sharp peaks, indicating the existence of phytochemical constituents that are either semi-crystalline or crystalline in nature [71]. Bhalodiya et al. (2021) used DSC to evaluate an ASD system with Boerhaavia diffusa extract [71]. In the research, the DSC thermogram for the pure extract showed a prominent endothermic peak at 115 °C. Between 115 °C and 210 °C, several endothermic and exothermic peaks were detected, and they were attributed to the presence of secondary metabolites. The DSC thermograms of the physical mixture containing all polymers reported that the phytochemical constituents in the extract retained a crystalline structure, and sharp endothermic peaks were seen [71]. In contrast, the ASD system was fully amorphous, since no identifiable crystalline peaks were shown in the thermogram [76]. The result of an ASD with Piper longum (PL) extract was also reported. The PL extract showed a prominent endothermic peak at 121.059 °C, indicating the presence of crystallinity, which is linked to the melting of various phytoconstituents. The Soluplus^®^, used as a polymer, exhibited a wide endothermic peak at 69.5 °C, indicating the glass transition temperature (Tg) and representing its amorphous nature. The physical mixture exhibited the typical endothermic peaks of PL extract and the polymer at ~121.2 °C and 68.8 °C, respectively. Nonetheless, the diminished intensity and slightly wider endotherm of the PL extract resulted from a higher content of the polymer. The thermograms of the ASD did not show the endothermic peak of the PL extract, indicating a complete transformation of the crystalline extract to an amorphous state. A broad endothermic peak was recorded at 50.239 °C, which was markedly different from Piper longum fruit ethanolic extract (PLFEE), PM, and Soluplus^®^. The Tg of Soluplus^®^ was shifted to a lower temperature of 50.2 °C, suggesting an interaction between the PL extract and the polymer [76].

### 7.3. Fourier Transform Infrared Spectroscopy (FTIR)

Fourier transform infrared spectroscopy (FTIR) has been extensively applied in the pharmaceutical industry to evaluate manufacturing processes concerning solid dispersions, phase separation, recrystallization stability, and the characteristics of drug–polymer interaction [12]. The FTIR measurement of an ASD with an extract was reported by Chen et al. (2020) [10,12]. The spectra confirmed the inclusion of PVP K-30 with distinct peaks, such as the C=O stretching of the pyrrolidone group at 1662.37 cm^−1^. The peak position shifted to 1657.56 cm^−1^ in ASD–SD as a result of hydrogen bonding. Similarly, FTIR analysis of pure extracts and their solid dispersions showed consistent characteristic peaks with broad OH stretching, CH3 groups, and carbonyl peaks at 3400 cm^−1^, 2900 cm^−1^, and 1728 cm^−1^. This showed the stability of the phytoconstituents without any adverse chemical impacts from the dispersion preparation methods. Solid dispersions with PVP K-30 showed FTIR spectra where the carbonyl peak shifted to higher wavenumbers and OH at 3400 cm^−1^ was suppressed, suggesting hydrogen bonding involving PVP K-30 and phytoconstituents [12]. In the formulation of ASD–ginkgo biloba extract (GBE), significant changes in C=O stretching bands were observed in the ASD system compared with physical mixtures, indicating distinct intermolecular interactions between GBE and the polymer. This research shows the utility of FTIR in probing molecular interactions and structural changes in solid dispersions, as well as determining the mechanisms of formulation stability and bioactive compound dispersion.

### 7.4. Powder X-Ray Diffraction (PXRD)

Powder X-ray diffraction (PXRD) is an essential tool for characterizing ASDs, and the method can be used to verify a drug’s presence in the amorphous form within a solid dispersion [85]. Wang et al. (2015) reported PXRD research on an ASD with GBE with spray-dried powder composed of Kollidon^®^ VA64 and Kolliphor^®^ RH40 at a weight ratio of 85:15 [12]. The pure GBE diffractogram showed characteristic peaks at 2θ = 7.34° and 2θ = 13.36°, while the physical mixture (PM) maintained crystallinity at a 25% load. Nonetheless, no clear intensity peaks were shown in the diffractograms of ASD formulations. These results confirm the transformation of GBE from a crystalline to an amorphous state in the ASD system [77]. Dudhat et al. (2023) conducted PXRD research to evaluate an ASD of Withania somnifera with the polymers Soluplus^®^ and PVP K-30. The PXRD diffractogram of the dried extract revealed prominent reflection peaks at angles of 16.29, 18.39, 19.18, 21.98, and 24.28, suggesting the existence of crystalline phytochemical components [17]. The diffractograms of the ASD formulated with Soluplus^®^ showed short and sharp diffraction peaks, where the transition of crystalline phytoconstituents into an amorphous form was incomplete. In contrast, the ASD prepared with PVP K-30 showed halos with no detected crystallinity, indicating the conversion of crystalline phytoconstituents into an amorphous form [17]. Measurements were also conducted by Xie et al. (2009) to evaluate an ASD of Hippophae rhamnoides L extract with poloxamer 188. The pure extract showed prominent characteristic peaks at a diffraction angle (2θ) of 9.02°, 9.78°, 13.35°, and 25.82° signifying the existence of a crystalline lattice of the drug. For pure poloxamer 188, characteristic peaks appeared at 19.17° and 23.33°. In the ASD system, the characteristic peaks for the crystalline pure extract were no longer present and were substituted by those of poloxamer 188, indicating the creation of an amorphous drug within the crystalline polymer matrix [77].

### 7.5. Thermogravimetric–Differential Thermal Analysis (TG-DTA) 

Thermogravimetric–differential thermal analysis (TG-DTA) is an essential tool for evaluating the loss of weight caused by degradation and the release of volatile components. This analysis determines the percentage of the total components [73]. Bennet et al. (2015) performed thermogravimetric analysis (TGA) to evaluate an ASD of gum resin extract. Acetyl-11-keto-b-boswellic acid (AKBA) obtained from gum resin extracts exhibited no weight loss before the melting point, indicating that the bulk API was thermally stable. The decomposition of the polymer was at 200 °C prior to the beginning of AKBA decomposition. Physical mixtures of AKBA and polymer were decomposed at 225 °C, and the recorded weight loss fell between the individual components of the drug and polymer. This phenomenon showed that the amorphous drug was formed during heating alongside polymers as a result of melting point depression [73]. Previous research also reported the use of TGA to evaluate ASDs. PLFEE showed a two-step degradation profile, initiating weight loss at 201.978 °C, suggesting thermal stability. A significant weight reduction of 64.2% was observed from 201.9 to 384.3 °C, followed by 17.3% from 384.3 to 800 °C. Meanwhile, the ASD showed an initial weight loss of 1.3% at 205 °C followed by 22.9%, 60.8%, and 13.9% from 205 to 344.0 °C, 344.0 to 467.3 °C, and 467.3 to 80 °C, respectively. The percentage weight loss in numerous steps differed from the others, indicating the presence of a completely new phase distinct from PM [85].

## 8. Dissolution Study

The formation of an ASD with a medicinal plant extract significantly enhances the dissolution rate. Jassim and AL-Khedairy (2023) reported the release of Silybum marianum (SM) extract in an ASD system [86]. The dissolution rate of SM from SD formulations was faster, with both achieving 100% release within 20 min [70]. The profiles of SD in an ASD system were also examined. As a result of the low solubility of the five major bioflavonoids obtained in a buffer with a pH of 6.8, less than 25% was released after 3 h. In the ASD system of SD, the release percentages for the five bioflavonoids were 95%. This was considered a complete release and was significantly higher than the SD. The results showed that ASD–SD achieved faster and higher dissolution rates than those of pure SD [76].

## 9. Pharmacological Activity

### 9.1. In Vitro Studies

Mohapatra et al. (2023) conducted an assay using PL extract in an ASD system [76]. The assay was conducted using the B16F10 melanoma cell line and HEK 293 human embryonic kidney cells. The result showed that ASD–PL extract enhanced the dose-dependent cytotoxicity against B16F10 cells in comparison with both the PL extract suspension and PL extract solution. The cytotoxicity of the ASD system against B16F10 melanoma cells was found to be significantly higher than that of the PL extract suspension and PL extract solution. Therefore, the ASD system enhanced the cytotoxicity activity of the PL extract. This higher cytotoxicity could be attributed to the elevated cell uptake and integration into cells. The free PL extract suspension also permeated the intracellular environment through passive diffusion. The poor solubility and resultant low concentrations of PL extract in the surrounding media led to decreased cellular absorption, contributing to low cytotoxicity. In contrast, the PL extract solution showed enhanced cytotoxicity due to a comparatively larger concentration in the medium and better diffusion. The ASD system improved the solubility in the surrounding medium and increased membrane permeability into the cell through the formation of micelles [81].

### 9.2. In Vivo Studies 

The improvement of the oral bioavailability of ASD–PL extract in vivo compared with that of a plain extract was reported by Mohapatra et al. (2023) [76]. The Cmax value of ASD–PL extract was approximately 2.21 times higher than that of ASD–PL. Moreover, the area under the curve of ASD–PL extract from 0 to 24 h was also significantly higher than that of ASD–PL. The mean residence time (MRT0-t) was lower than that of the plain extract, indicating a faster elimination of ASD. Meanwhile, the relative bioavailability (Frel) was determined to be 188.765%, which was significantly greater than that of the pure PL extract. The effect of the ASD system with the PL extract was analyzed in a syngeneic transplantation model involving C57BL/6 mice with melanoma (B16F10). The in vivo tumor regression showed that the ASD system improved therapeutic activity compared with the plain PL extract. The system also enhanced the anticancer effectiveness of dacarbazine (DTIC) in its role as an adjuvant therapy [76]. Yu et al. (2017) reported the average arterial plasma concentration–time profiles of baicalein obtained from RS extract following an oral dose of 15 mg/kg in rats [82]. Meanwhile, the plasma levels of baicalein were increased following oral administration compared with the powder, leading to significant increases in AUC24h, AUCINF, and Cmax by 40.3%, 44.1%, and 284.9%, respectively. The mean percentage of the oral dose of baicalein excreted in urine at 24 h was also substantially higher following oral administration of the solid dispersion formulation (23.0% vs. 28.7%). Absorption of baicalein from the rat’s gastrointestinal tract appeared to occur quickly, reaching peak plasma concentration at 15 min post-administration. However, a significantly delayed peak plasma concentration was observed after oral administration of the solid dispersion formulation. These results showed that the solid dispersion technique could enhance the bioavailability of baicalein [77].

## 10. Discussion

The incorporation of bioactive molecules into ASD systems is essential for improving the solubility, dissolution rate, and bioavailability of compounds that are poorly soluble. These systems have received significant attention, especially for natural extracts that are abundant in phytochemicals. The main objective of ASD development is to convert crystalline active chemicals into an amorphous state. This transformation is crucial for multicomponent extracts, guaranteeing the solubilization of diverse phytoconstituents that possess varying solubility and dissolution properties. The incorporation of extracts into ASD systems enhances the solubility and stability of individual components to facilitate the uniform dispersion of bioactive chemicals.

Different characterization and assessment methods are performed to validate the successful development of ASDs and analyze the ability to enhance the pharmacological activity of the loaded extracts. Methods such as SEM, X-ray diffraction (XRD), DSC, TGA, FTIR, solubility and dissolution tests, and in vitro/in vivo pharmacological investigations are essential for assessing the characteristics and effectiveness of these systems.

SEM offers significant insights into the morphological alterations caused by ASD development. Examinations of multicomponent extracts showed the transition from crystalline to amorphous structures, which are often characterized by a smoother surface morphology and a lack of crystalline particles. This morphological change indicates a successful transition to the amorphous state to enhance solubility.

DSC and XRD are complementary methods used to validate the amorphous characteristics of ASDs. In this context, DSC obtains data on thermal transitions, including melting points and glass temperatures. Meanwhile, XRD serves as a direct method for assessing the crystalline or amorphous state of a material. The absence of distinct diffraction peaks in XRD spectra with the lack of endothermic peaks in DSC thermograms unequivocally signifies successful amorphization. Therefore, the crystalline bioactive chemicals are completely integrated into the polymer matrix to improve solubility and stability.

FTIR is used to evaluate molecular interactions within ASDs, and the existence of hydrogen bonding or other non-covalent interactions can be validated by shifts in characteristic absorption bands. These interactions sustain the amorphous state and inhibit recrystallization to preserve the enhanced dissolving profile of ASDs. However, obtaining a specific compound from the extract interacting with the polymer is difficult due to the multiple compounds in the extracts. 

TGA offers insights into the thermal stability of ASD formulations. Such an analysis aids in identifying deterioration trends and evaluating the thermal stability of multicomponent extracts. An effective ASD formulation must show negligible weight loss at high temperatures, signifying that the encapsulated substances are resistant to early breakdown. Research on solubility and dissolution further validates the advantages of ASDs. The elevated dissolution rates in ASD-loaded extracts show the efficacy of amorphization in improving the release of bioactive chemicals. In this context, pure extract formulations show a substantially faster and more complete solubility than that of ASDs, and this is directly correlated with enhanced bioavailability and pharmacological activity.

In vitro and in vivo research is essential for converting the physicochemical enhancements in ASD formulations into tangible pharmacological advantages. Generally, increased solubility and dissolution rates lead to elevated plasma concentrations, enhanced absorption, and superior therapeutic results. This confirms the pharmacokinetic enhancements provided by ASD systems, including elevated Cmax and AUC values, which show improved bioavailability. Moreover, in vitro investigations of bioactive substances frequently show enhanced efficacy in cellular models, substantiating the possibility of augmenting therapeutic activity.

**Figure 2:**
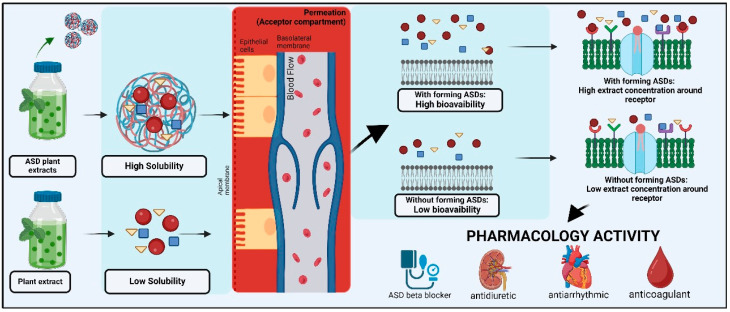
The speculated mechanism of the improvement of the bioavailability of plant extracts in ASD systems.

The mechanism used to enhance pharmacological action is due to the augmented solubility rate and bioavailability of the bioactive chemicals, as shown in Figure 2. An ASD improves solubility by converting crystalline phytoconstituents into amorphous forms, leading to enhanced absorption in biological systems. Additionally, the interaction between bioactive chemicals and the polymer matrix inhibits early breakdown, leading to sustained release and extended activity. The absorption of extracts is enhanced when phytoconstituents are in a supersaturated state. Previous research reported that a drug’s solubility in an aqueous compartment significantly affected the amount of absorption through passive diffusion. Therefore, the mechanism of phytoconstituent absorption occurs through passive diffusion, where the soluble phytoconstituents in the aqueous compartment, like those in the interstitial space, migrate to the endothelium of blood vessels. The absorption of phytoconstituents is increased due to enhanced solubility and the maintenance of a supersaturated solution through intermolecular interactions. Higher concentrations of molecularly dissolved phytoconstituents significantly enhanced permeation rates, leading to the bioavailability of phytoconstituents. Improving the bioavailability of plant extracts or phytoconstituents also enhances their pharmacokinetics, efficacy, and safety. However, significant constraints exist in the examination of multicomponent extracts inside ASD systems. The intricate nature of natural extracts, which comprise several active and inert constituents, presents difficulties for characterization and assessment. Determining the precise contribution of each chemical inside an ASD matrix is challenging due to the variable interactions among the different ingredients and polymers. This intricacy frequently affects the interpretation of results from analytical methods such as DSC, FTIR, and XRD, since several overlapping peaks or signals may mask definitive conclusions. Moreover, the scalability and reproducibility of ASD formulations for complex extracts pose a problem due to the diversity in the composition of natural extracts.

Even though ASDs offer a beneficial opportunity to enhance the solubility and bioavailability of multicomponent extracts, additional research is necessary to tackle the issues associated with the complexity of such systems. The capacity to enhance the pharmacological profile of bioactive chemicals renders ASDs an invaluable asset in the production of efficacious therapeutic agents derived from natural extracts.

## 11. Conclusions

In conclusion, ASDs containing multicomponent chemicals from plant extracts significantly enhance the bioavailability and pharmacological efficacy of naturally derived substances. The use of analytical methods such as SEM, XRD, DSC, TGA, and FTIR enables the examination of the structural and chemical properties of ASDs, providing essential insights into the stability and molecular dynamics. Moreover, solubility investigations and in vitro/in vivo assessments further validate the ability of ASDs to address solubility and bioavailability issues. The intricacy of multi-component systems, especially in natural extracts, presents significant obstacles to characterization and reproducibility. Meanwhile, the interconnectedness of diverse chemicals, probable degradation during processing, and intricate interaction patterns necessitate meticulous investigation to properly leverage the benefits of ASDs. The increasing interest in natural goods and the demand for enhanced pharmacokinetics render ASDs a potential and problematic pathway for pharmaceutical progress. This duality originates from ASDs’ capacity to efficiently tackle solubility and bioavailability issues while concurrently posing hurdles in regulating the intricate interactions, stability, and scalability of natural multicomponent systems. Even though ASDs are a revolutionary method for harnessing the therapeutic advantages of multicomponent extracts, their complete potential is only achieved through meticulous characterization, formulation optimization, and in vitro and in vivo validation. These investigations are crucial in overcoming the challenges of solubility, stability, and bioavailability to enhance ASD systems as a dependable drug delivery platform.

## Figures and Tables

**Table 1:** Pharmacological activity of medicinal plant extracts.

No.	Extract	Active Substance	Pharmacological Activity	Ref.
1.	*Selaginella doederleinii* extract (SD)	Phytone, cedrol, 2-pentylfuran, caryophyllene, n-hexadecane	Antiviral, anti-inflammatory, anticancer, antioxidant, antihyperuricemic, antibacterial, antihypertensive, and potential therapeutic properties in handling the symptoms of Alzheimer’s illness	[23]
2.	Punarnava extract (*Boerhaavia diffusa* (Linn.)) (BD)	Phenolic, rotenoid, flavonoid, isoflavonoid, alkaloid, steroid, anthracenes, lignans	Anti-aging, antioxidant, antifungal, antibacterial, anticancer, anti-inflammatory, antidiabetic, hepatoprotective, cardioprotective, renoprotective, antifertility	[24]
3.	Ashwagandha extract (*Withania somnifera*) (WS)	Polyphenols, ascorbic acid, flavonoid	Antioxidant, antibacterial	[25]
4.	*Epigallocatechin gallate* extract (EGCG)	Flavone-3-ol polyphenol	Antioxidant, anti-inflammatory, anticancer, antibacterial	[26]
5.	Gum resin (*Boswellia*)	Alpha-pinene, a-boswellic acid, acetyl-a-boswellic acid, 11-keto-b-boswellic acid, lupeolic acid, 3-O-acetyl-a-boswellic acid, acetyl-b-boswellic acid, acetyl lupeolic acid	Immunomodulatory, anti-inflammatory, hypoglycemic, anticancer, anti-asthmatic, antidiarrheal, hypolipidemic, anti-diabetic, hepatoprotective, and even antiviral	[27]
6.	*Ginkgo biloba* extract (GB)	Terpenoids, flavonoids, carboxylic acids, lignins, proanthocyanidins, polyprenols, polysaccharides, alkylphenols, alkylphenolic acids	Anti-inflammatory, antimicrobial, antioxidant, anti-tumor, anti-obesity, anti-atherogenic, anti-diabetic, neuroprotective, anti-neurodegenerative, protection of other organs	[28]
7.	Knotweed extract *(Polygonum cuspidatum)* (PC)	Flavonoids, anthraquinones, stilbenes	Anti-inflammatory, antioxidation, neuroprotection, hepatoprotective, cardioprotective, blood vessel protective, antiviral, antibacterial, antifungal, anticancer, heart protection	[29]
8.	*Kaempferia parviflora*	Ethanol extract, 7,4-trimethoxyflavone, 3,5,7,4′-tetramethoxyflavone, 5,7,4′- trimethoxyflavone and 5-hydroxy3,7,3′,4′-tetramethoxyflavone	Anticancer, anti-tumor, antibiotics, antimicrobial	[30,31]
9.	*Piper longum*	Amide alkaloids	A-549 (human lung cancer), SMMC-7721 (human liver cancer), SW480 (human rectal cancer) exhibit various biological activities, including anti-hepatitis B virus (antiHBV), apoptotic, leishmanicidal, acylCoA: cholesterol acyltransferase (ACAT) inhibitory, cytotoxic, mosquito larvicidal, MCF-7 (human breast cancer), phytotoxic, anti-inflammatory, HL-60 (human leukemia), antihyperlipidemic, cell adhesion inhibitory, antiplatelet, antifungal, and coronary vasorelaxant effects	[32]
10.	*Toxicodendron vernicifluum*	Flavonoids, phenolic acids, and their derivatives, catechin concoction-urushiols, and tannins	Anti-inflammatory activity, anti-arthritic activities, immunomodulatory activity, anticancer activity	[33]
11.	*Hippophae rhamnoides* L.	Vitamins, amino acids, fatty acids, carotenoids, phenolic	Anti-inflammatory, antifungal, antioxidant, spasmolytic, antihistaminic, gastroprotective, antiviral, anticarcinogenic, cardioprotective, antibacterial, and radioprotective effects	[34]
12.	*Curcuma longa*	Terpenoids and phenolic	Anti-inflammatory, antioxidant, and anticancer	[35]
13.	*Moringa oleifera* leaf	niazirin, kaempferol-3-O-(6″-malonyl-glucoside), niazirinin, flavonoid, anthocyanin, proanthocyanidin, 4-hydroxycinnamic acid, and β-sitosterol	Anticancer, hepatoprotective, antioxidants, antidiabetic, antimicrobial, and menopause inhibition	[36]
14.	*Centella asiatica*	madecassic acid, asiaticoside, asiatic acid, and madecassoside	Cardioprotective, antiatherosclerotic, antihypertensive, antihyperlipidemic, anti-diabetic, antioxidant, and anti-inflammatory	[37]
15.	*Red ginseng*	More than 40 ginsenosides	Enhancing immunity, improving memory, anti-tumor, anti-aging, anti-radiation, and anti-fatigue	[38]
16.	*Ginger*	Phenolic compounds and terpenes	Antimicrobial, antioxidant, anti-inflammatory, anticancer, and anti-allergic properties related to various central nervous system functions	[35]
17.	*Radix scutellariae*	Phenols, flavonoids, terpenoids, tannins, alkaloids, and saponins	Antiviral, antibacterial, anti-inflammatory, and antioxidant properties, with potential applications in the treatment of periodontitis	[39]
18.	*Prosopis africana*	The amino acids lysine, leucine, alanine, proline, methionine, phenylalanine, cysteine, and threonine, along with vitamins A, B2, B5, B6, B9, and B12	Anti-helminthic, antioxidant, anti-inflammatory, antifungal, anti-microbial, immunostimulatory, and hepatoprotective abilities	[40]
19.	*Silybum marianum*	Flavonolignans	Antioxidant, anti-inflammatory, and neuroprotective properties	[41]

**Table 2:** The studies of ASD system from medicinal plant extracts.

No.	Extract	Polymer	Method	Characterization	Dissolution Research	Pharmacology Activity		Ref.
						In Vitro	In Vivo	
1.	*Selaginella doederleinii*	PEG 6000 (Polyethylene glycol 6000), PEG 4000 (Polyethylene glycol 4000), Poloxamer 188, and PVP K-30 (Polyvinylpyrrolidone K-30)	Solvent evaporation	SEM (Scanning electron microscopy), DSC (Differential scanning calorimetry), PXRD (Powder X-ray diffraction), FTIR (Fourier transform infrared spectroscopy)	The release rate of all five components in an ASD (amorphous solid dispersion) with an extract was markedly superior to that of the raw extract.	-	Oral administration of ASD with extract to xenograft-bearing tumor mice resulted in a notable decrease in tumor size and microvascular density compared with the raw extract.	[70]
2.	*Boerhaavia diffusa* (Linn.)	HPMCAS-L (Hydroxypropyl methylcellulose acetate succinate grade low) and PVP K-30	Solvent evaporation	DSC, PXRD	The findings of the saturation solubility study demonstrated a notable enhancement in the solubility of the phytoconstituent (at ratios of 1:2 and 1:4) in comparison with the pure extract.	-	-	[71]
3.	*Withania somnifera*	PVP K-30	Solvent evaporation	DSC, PXRD, FTIR	The prepared ASD has significantly higher saturation solubility and dissolution than the pure extract.	-	-	[17]
4.	*Epigallocatechin Gallate*	Soluplus^®^	Lyophilization	XRPD (X-ray powder diffraction)	The Soluplus^®^ dispersion of extracts demonstrates a markedly sustained release profile in comparison with pure extracts.	-	-	[72]
5.	*Gum resin*	HPMCAS (Hydroxypropyl methylcellulose acetate succinate)	KinetiSol Dispersing (KSD) and Rotary evaporation (RE)	TGA (thermogravimetric analysis), XRD (X-ray diffraction), SEM,	The presence of polymer in the ASD system enhanced the dissolution properties of the extract in comparison with pure extract.	-	The ASD with an extract demonstrated enhanced bioavailability in an animal model relative to the administered commercial formulation of the raw extract.	[73]
6.	*Ginkgo biloba* (GBE)	Kollidon^®^ VA64 (Vinylpyrrolidone-vinyl acetate copolymers) and Kolliphor^®^ RH40 (Polyoxyl 40 castor oil), in a weight ratio of 85:15 (*w*/*w*), are processed into a spray-dried powder.	Hot-melt extrusion	DSC, PXRD, FTIR	ASD with GBE (Amorphous solid dispersion of *Ginkgo biloba*) showed faster dissolution rates and higher dissolution compared with GBE and PM (physical mixture).	-	The pharmacokinetic profiles and parameters demonstrated that the Cmax (maximum serum concentration) and AUC0−t (area under the concentration–time curve from time zero to the last measurable concentration) of the active ingredients could be markedly improved following the formulation of solid dispersion.	[12]
7.	*Polygonum cuspidatum* (PC)	HPMCAS and P188 (Poloxamer 188)	Hot-melt extrusion	FTIR, SEM, XRD	ASD PC (amorphous solid dispersion of *Polygonum cuspidatum*) extract increased the dissolution from 46.75 ± 0.47% to 130.06 ± 0.12%.		An ASD with an extract significantly improved the oral bioavailability compared with the pure extract.	[74]
8.	*Kaempferia parviflora (KPD)*	HPMC (Hydroxypropyl methylcellulose) PVA-co-PEG (Co-polymerization of polyvinyl alcohol with polyethylene glicol)	Solvent evaporation	DSC, PXRD	Dissolution of a selected marker, 5,7,4′-trimethoxyflavone (TMF), from ASD- KPD/HPMC (amorphous solid dispersion of *Kaempferia parviflora* with HPMC) and ASD-KPD/PVA-co-PEG (amorphous solid dispersion of *Kaempferia parviflora* with PVA-co-PEG) was significantly improved compared with pure KPD.	-	-	[75]
9.	*Piper longum* (PL)	Soluplus^®^	Solvent evaporation	DSC XRD	The in vitro dissolution research revealed excellent wetting of ASD–PL (amorphous solid dispersion of *Piper longum*) extract and an improved dissolution profile compared with plain PL.	-	The in vivo tumor regression research revealed the improved therapeutic activity of ASD–PL extract compared with plain PL.	[76]
10.	*Hippophae rhamnoides* L (TFH)	poloxamer 188 (PXM)	Solvent evaporation	FTIR, DSC, PXRD, SEM	The dissolution enhancement of TFH was observed in ASD–TFH (amorphous solid dispersion of *Hippophae rhamnoides* L) compared with pore TFH and a physical mixture.	-	-	[77]
11.	*Curcuma longa* (CL)	PVP/VA (Vinylpyrrolidone/vinyl acetate copolymers)	Solvent evaporation	DSC	The dissolution rates of solid dispersions were better than those of the curcuma extract.	-	ASD–CL (amorphous solid dispersion of *Curcuma longa*) was able to improve the intestinal absorption of the curcuma extract compared with pure CL.	[78]
12.	*Centella asiatica* (CA)	Eudragit^®^ EPO (Eudragit for pharmaceutical operation),	Solvent evaporation	PXRD, FTIR	The dissolution of AS (Asiaticoside) and MS (Madecassoside) from solid dispersions of CA was more rapid, resulting in complete release within 10 min compared with the CA extract.	.-	ASD–CA (amorphous solid dispersion of *Centella asiatica*) reduced the severity of gastric ulcers induced by indomethacin with a greater curative efficacy than that of unformulated CA extract and a standard antiulcer agent: lansoprazole.	[79]
13.	*Red ginseng* (RG)	PVP, HPC (Hydroxypropyl cellulose), PVPc (Cross-linked polyvinylpyrrolidone), P6000 (Polyethyleneglycol 6000), P1500 (Polyethyleneglycol 1500), and silicon dioxide	Solvent evaporation	XRD, DSC	-	-	The formulation of ASD-RG (amorphous solid dispersion of red ginseng) improved hygroscopicity, increased intestinal permeability, and enhanced oral bioavailability.	[80]
14.	*Ginger*	PVP K-30 and Eudragit EPO	Solvent evaporation	X-ray, FTIR, SEM	ASD–ginger extract exhibited a high rate of drug release.	-	ASD–ginger showed dose-dependent cytotoxic activity against AGS human gastric adenocarcinoma cells and anti-inflammatory activity.	[81]
15.	*Radix scutellariae* (RS)	PVP K-30	Solvent evaporation	DSC, X-ray, FTIR, SEM	ASD–RS (amorphous solid dispersion of *Radix scutellariae*) showed a significantly higher release rate than the control (pure RS) due to high solubility.	-	Pharmacokinetic evaluation of baicalein obtained in RS extract showed that the bioavailability of baicalein could be significantly improved by an ASD due to increased solubility and dissolution rate.	[82]
16.	*Prosopis africana* (PA)	PEG 4000	Melt fusion	FTIR, DSC	The presence of polymer in ASD–PA (amorphous solid dispersion of *Prosopis africana*) improved the dissolution profile of the PA extract.	-	-	[83]

## Data Availability

Not applicable.
